# Predictors of Quality of Life in Parkinson's Disease: The Role of Mental Health and Internalized Stigma

**DOI:** 10.62641/aep.v54i1.2130

**Published:** 2026-02-15

**Authors:** Ana Fresan, Adriana Ochoa-Morales, Miguel Ángel Ramírez-García, Mireya Chávez-Oliveros, Aurelio Jara-Prado, Jorge Luis Guerrero-Camacho, David José Dávila-Ortiz de Montellano

**Affiliations:** ^1^Biomedical Research in Mental Health Directorate, Ramón de la Fuente Muñiz National Institute of Psychiatry, 14370 CDMX, Mexico; ^2^National Institute of Neurology and Neurosurgery, Manuel Velasco Suárez, 14269 CDMX, Mexico

**Keywords:** Parkinson's disease, internalized stigma, caregiver burden, depression, anxiety

## Abstract

**Background:**

Parkinson's disease (PD) is a progressive neurodegenerative disorder that negatively affects the well-being of both patients and their caregivers. Therefore, the aim of the present study was to identify factors associated with quality of life (QoL) in patients with Parkinson's disease (PPD) and caregiver burden in their primary caregivers (PCG).

**Methods:**

We conducted a cross-sectional study at a tertiary neurological center in Mexico. Assessments included the Parkinson's Disease Questionnaire (PDQ-39); motor severity with the Movement Disorder Society–Unified Parkinson's Disease Rating Scale Part III (MDS-UPDRS III); cognition with the Montreal Cognitive Assessment (MoCA); depressive and anxiety symptoms with the Patient Health Questionnaire-9 (PHQ-9) and the Generalized Anxiety Disorder-7 (GAD-7); and internalized stigma with the King Internalized Stigma Scale (ISS). Caregiver burden was measured with the Zarit Burden Interview (ZBI).

**Results:**

We included 48 PPD (58.3% male) and 38 PCG (55.3% female). Mean disease duration was 7.3 years [standard deviations (SD) = 4.6; range 1–26 years]. Among PPD, 97.9% were on dopaminergic replacement therapy and 43.8% reported comorbidities. Anxiety severity differed between groups (χ^2^ = 11.7, *p* = 0.008). No between-group differences were observed in internalized stigma (ISS total score and subdomains). A significant discrepancy emerged regarding assistance with activities of daily living (ADLs), reported by 63.2% of PCG versus 20.8% of PPD (*p* < 0.001). Linear regression models showed that poorer QoL in PPD was associated with depressive and anxiety symptoms and motor severity (MDS-UPDRS III) (R^2^ = 0.47). Caregiver burden in PCG was associated with depressive symptoms and perceived discrimination (ISS subdomain) (R^2^ = 0.53).

**Conclusions:**

Comprehensive PD management, beyond motor control, should incorporate the evaluation and support of mental health, alongside stigma-reduction strategies, to enhance the well-being of both PPD and their PCG.

## Introduction

Parkinson’s disease (PD) is a neurodegenerative disorder whose prevalence increases with age and is currently considered among the fastest-growing neurological diseases in the world [1]. It is the second most prevalent neurodegenerative disease worldwide [2]. In developed countries, the prevalence of PD is estimated to be 0.3% of the total population and about 1% in individuals over 60 years of age [3]. Globally, the burden of PD continues to rise; using Global Burden of Disease (GBD) 2023 data, PD was estimated at 11.67 million prevalent cases worldwide with an age-standardized prevalence rate (ASPR) of 128.5 per 100,000 and an age-standardized incidence rate (ASIR) of 15.11 per 100,000 [4]. At the national level, the highest age-standardized rates reported for 2021 were in China (ASIR 24.34/100,000 and ASPR 245.73/100,000) [2]. In Latin America, pooled estimates summarized alongside GBD 2023 show wide variation, with prevalence reported as high as 1081 per 100,000, in contrast to substantially lower values in other regions [5]. In GBD 2023 country estimates, Mexico shows an ASPR of 182.5/100,000 (female 192.7; male 175.5), compared with higher ASPR estimates in Brazil (284.0/100,000) and Paraguay (291.7/100,000) [4, 5]. Although precise national surveillance statistics are limited, a nationwide administrative-data analysis in Mexico estimated an incidence of 9.48 per 100,000 person-years among adults >20 years during 2014–2019, projecting an increase to 14.9 per 100,000 by 2023, with the highest incidence in Sinaloa (27.6/100,000), Colima (23.5/100,000), and Durango (20.0/100,000), potentially linked to environmental exposures such as pesticides and contaminated water [6]. PD is characterized by motor symptoms, including rigidity, bradykinesia, tremor, and postural instability [7], and non-motor symptoms, including gastrointestinal, autonomic, and neuropsychiatric disturbances (including depression, anxiety, cognitive impairment, sleep disturbances, apathy, slow thinking, and psychosis) [8]. Another recognized problem in patients with Parkinson’s disease (PPD) is difficulty with cognitive control [9]. Among mental health problems, depression and anxiety are the most common neuropsychiatric complications of PD, with predisposing factors including early onset, advanced stage, persistent body rigidity, and female sex [10, 11].

As the disease progresses and clinical manifestations accumulate, both PPD and their PCG face challenges related to adapting to changes in family roles, loss of employment, stigma, and increasing physical and mental disability, which negatively impact PPD quality of life (QoL) [12, 13, 14]. In addition to these, primary caregivers (PCG), who play a crucial role in patient care [15], face the economic consequences of treating and caring for someone with a neurodegenerative disease, and their daily activities may be affected. Collectively, these experiences and daily life changes may contribute to greater PCG burden. Hence, multidisciplinary care for both PPD and their PCG is crucial to facilitate optimal coping strategies for both.

Several factors influence the perception of QoL in PPD and burden in PCG, including symptom control, adherence to treatment, psychiatric symptoms, such as depression and anxiety, and perceived social support [16]. However, other factors—such as stigma—may also exert a negative influence.

Historically, neurological and neuropsychiatric disorders have been among the most stigmatized conditions, and PD is no exception. The concept of stigma refers to either the societal reaction to various conditions (externalized stigma) or the self-devaluation of individuals (internalized stigma) through the internalization of negative stereotypes about themselves or their social group or the disease [17]. The latter is considered both a risk factor and an indicator of poor prognosis for mental health and social behavior [18]. Because of the neurological and emotional symptoms that the disease produces, PPD often feel abandoned, discriminated against, and/or stigmatized [19], as do their families. Family members of people with Parkinson’s disease may internalize stigma due to ongoing exposure to negative social reactions and through identification with the visible limitations of the patient, which can foster feelings of shame, guilt, and a sense of “courtesy”. Furthermore, caregiving burden, limited public understanding of non-motor symptoms (e.g., incontinence, depression), and culturally shaped beliefs about disease and disability may facilitate the internalization of external stigmatizing attitudes into caregivers’ own beliefs [20, 21]. Therefore, this study aimed to determine whether clinical characteristics (cognition, severity of depression and anxiety), sociodemographic factors, and stigma are associated with QoL in PPD and burden in PCG.

## Methods

A cross-sectional study was conducted and approved by the Research and Ethics Committees of the National Institute of Neurology and Neurosurgery Manuel Velasco Suárez, Mexico (Approval No. 33/22) (INNNMVS) and all procedures were in compliance with the Declaration of Helsinki. All participants received a verbal explanation of the study objectives and procedures. Those who expressed interest were provided with the informed consent form, had any questions answered, and, upon confirming their willingness to participate, signed the informed consent.

Patients attending the INNNMVS come from different regions across the country. As a national referral center, the INNNMVS provides specialized neurological care and attracts patients with complex or treatment-resistant conditions from various states of Mexico, ensuring a diverse and representative clinical population. Participants in this study were recruited between January 2023 and June 2024. They participated voluntarily, were not compensated for their participation, and could withdraw their consent at any time during the evaluation without any consequences for their clinical care.

### Sample

The a priori sample size calculation was conducted, assuming an expected *d* effect size of 0.7 [22], with an alpha error of 0.10 and a statistical power of 0.90. Based on these parameters, it was estimated that each group should include at least 36 participants. The calculation was performed using the G*Power software, version 3.1.9.7., Heinrich-Heine-Universität Düsseldorf, Germany.

The inclusion criteria for the patient group required a diagnosis of Parkinson’s disease, confirmed by treating physicians through clinical evaluation and MDS (Movement Disorder Society) criteria [23, 24], and treatment at the Movement Disorders Clinic of the INNNMVS. Participants with clinically documented dementia or other motor/cognitive impairments that precluded valid completion of the assessments were excluded. To minimize confounding by established psychiatric disease, participants with documented depressive or anxiety disorders in the medical record were also excluded. Nevertheless, PHQ-9, GAD-7, and MoCA were administered to characterize current symptom severity and screening-level cognitive performance at the time of evaluation, which may include subclinical findings. No patients withdrew their consent to participate. For the family group, inclusion criteria required being the non-professional primary caregiver of a patient with Parkinson’s disease, attending the medical follow-up visit with their relative, and being able to read and write. Family members with any self-reported neurological, psychiatric, or medical condition that could interfere with the proper completion or understanding of the study instruments were excluded.

The present study included 48 PPD who had a clinical diagnosis confirmed by a neurologist specializing in movement disorders at the outpatient clinic of the INNNMVS and 38 PCG who accompanied the PPD. As some PPD attended appointments alone, the groups were unequal in size.

### Instruments

Assessments were conducted on a single occasion for both PPD and PCG. These assessments took place after their medical visit at the Movement Disorders Clinic and once eligible candidates had agreed to participate in the study and provided written informed consent.

The QoL of PPD was assessed using the Parkinson’s Disease Questionnaire (PDQ-39) [25, 26], which consists of 39 Likert-type items, scored from 0 (never) to 4 (always), with higher scores indicating lower QoL. It is divided into 8 domains: mobility, activities of daily living, emotional well-being, stigma, social support, cognition, communication, and bodily discomfort. Internal consistency of the instrument was satisfactory (Cronbach’s alpha values ranging from 0.63–0.94) and adequate convergent validity was reported for its use [26].

Cognition was assessed using the Montreal Cognitive Assessment (MoCA), a cognitive screening test for the detection of mild cognitive impairment (MCI). It consists of 30 items assessing memory, visuospatial ability, executive function, attention/concentration, language, and orientation. Scores range from 0 to 30, with a cutoff score of 26, indicating adequate sensitivity and specificity for detecting MCI [27, 28]. A reliability coefficient of 0.89 and an intraclass correlation coefficient of 0.95 were obtained for the MoCA-S. It also demonstrated adequate discriminant validity among control subjects, individuals with mild cognitive impairment, and those with dementia [28].

Depressive and anxiety symptoms were assessed using the Patient Health Questionnaire-9 (PHQ-9) and the Generalized Anxiety Disorder-7 (GAD-7) scales, respectively. Both are self-administered Likert-type scales with higher scores indicating greater symptom severity. Cutoff scores for both instruments are 0–4 (none or minimal symptoms), 5–9 (mild symptoms), 10–14 (moderate symptoms), 15–19 (moderately severe) and ≥20 (severe symptoms) [29, 30, 31, 32]. The PHQ-9 demonstrated adequate construct validity as well as proper reliability with a Cronbach’s alpha >0.80) [32]. Although no published studies have formally validated the GAD-7 specifically in Mexican older adults, the instrument has demonstrated acceptable psychometric properties in general Mexican populations (adequate factor solution for construct validity, internal consistency and intraclass correlation coefficient) [33].

The King Internalized Stigma Scale (ISS) is a 28-item scale scored on a five-point Likert scale, ranging from 0 (strongly disagree) to 4 (strongly agree). It assesses internalized stigma across three domains: discrimination, disclosure, and positive aspects of the disease, as well as general internalized stigma (ranging from 0 to 112 points) [34, 35]. The total score is obtained by summing the scores of each domain, with higher scores reflecting greater internalized stigma. The scale demonstrated adequate construct validity as well as satisfactory internal consistency in the validation study performed with Mexican subjects [35].

The Movement Disorder Society–Unified Parkinson’s Disease Rating Scale Part III (MDS-UPDRS III) [36] was applied by clinicians of the Movement Disorders Clinic and used to assess the disease state of PPD. The total scale score ranges from 0 to 132 points, with higher scores indicating greater disease severity, where the maximum score reflects total disability [37, 38]. Concurrent validity of the scale was determined as adequate (correlation coefficient of 0.96) as well as its reliability, with values ranging from acceptable to excellent (0.79–0.93) [36]. Finally, PCG burden was assessed using the Zarit Burden Interview (ZBI), a self-administered Likert-type scale ranging from 0 to 88. A score below 40 indicates no burden, 41–60 indicates mild burden, and above 61 indicates severe PCG burden [39, 40, 41]. Adequate values of construct validity and internal consistency for reliability were obtained in Mexican population [40].

### Statistical Analysis

Frequencies and percentages were used for categorical variables, while means and standard deviations (SD) were used for continuous variables. The distribution of the study variables showed acceptable univariate normality, with skewness values ranging between –1.10 and +1.35 and kurtosis values between –1.0 and +1.4, indicating that assumptions for parametric analyses were met. Therefore, chi-square tests and independent *t*-tests were conducted for group comparisons between PPD and PCG participants. In addition, linear regression models with backward stepwise selection were performed for PPD and PCG to determine factors associated with QoL, with variable retention set at *p* ≤ 0.05. Variance inflation factor (VIF) values were determined to measure multicollinearity; any variable with a VIF value greater than 10 was excluded from the regression analyses. For both groups, demographic characteristics (sex, age, years of education) and psychiatric symptoms (severity of depressive, anxious, and cognitive symptoms) were included in the first model, but as no significant values were obtained, they were not included in the final modelling. In the PPD group, only clinical characteristics (disease duration, MDS-UPDRS III), internalized stigma (ISS), and patient QoL were included, with QoL as the dependent variable. For the PCG group, the total score on the ZBI (PCG burden) was the dependent variable, in addition to PCG characteristics (hours of caregiving) and internalized stigma (ISS). The post hoc statistical power of the linear regression analysis, given the number of participants recruited, was 0.90, considering the number of independent variables included in each model. This analysis was performed using the G*Power software, version 3.1.9.7., Heinrich-Heine-Universität Düsseldorf, Germany.

Statistical analyses were performed using SPSS version 26 for Windows PC, IBM corp, Armonk, NY, United States. Statistical significance was set at *p* < 0.05.

## Results

### Demographic and Clinical Characteristics

A total of 48 PPD (58.3% male) and 38 PCG (55.3% female) were included. PCG were significantly younger than PPD, with no differences in years of education between the groups. PPD showed a higher proportion of unemployment than PCG, without reaching statistical significance.

The mean MDS-UPDRS III score was 28.3 points, indicating mild motor impairment [37] among the patients evaluated in the present study. The mean duration of disease was 7.3 years (SD = 4.6, range 1–26 years). In our cohort, all patients were managed according to standard-of-care pharmacological treatment tailored to their clinical characteristics. Consistent with current evidence-based recommendations for motor fluctuations—where patients are typically already receiving levodopa and oral therapy is optimized before escalation—most PPD were on dopaminergic replacement therapy (DRT) at the time of the study (n = 47, 97.9%) [42], and 43.8% (n = 21) reported comorbidities, most commonly hypertension (n = 12, 57.1%) or diabetes mellitus (n = 9, 42.9%).

The caregivers’ relationship with patients with Parkinson’s disease was heterogeneous. Most caregivers were the patient’s partner (34.2%, n = 13), son or daughter (34.2%, n = 13), or a sibling (23.7%, n = 9). The remaining caregivers included a grandchild, a parent, and a friend.

Socioeconomic status among participants was predominantly middle (n = 25, 58.3%) and low (n = 19, 39.6%), with no statistically significant differences between groups.

In addition, primary caregivers dedicated a mean of 9.2 hours per day to caregiving (SD = 6.9), with a range of 1 to 24 hours. More than sixty percent of PCG (n = 24, 63.2%) reported that the person they cared for needed help with activities of daily living (ADLs). In contrast, only 20.8% (n = 10) of PPD reported that they required a PCG to assist them with those activities (*p* < 0.001). The mean score on the ZBI was 21.6 (SD = 17.1; range 0–60), indicating low average burden, with wide variability reported by PCG. Socioeconomic status (SES) was recorded for PPD when available in clinical records; however, SES data were not available for PCG, precluding between-group comparisons. Socioeconomic status was predominantly middle (n = 25, 58.3%) and low (n = 19, 39.6%) among PPD. Table 1 summarizes the remaining characteristics and between-group comparisons for PPD and PCG.

**Table 1.** **Demographic and clinical features between patients with Parkinson’s disease and primary caregivers**.

		Total	Patients	Primary caregivers	Statistics
		n = 86	n = 48	n = 38	
Demographic variables					
Sex*§*					
	Woman	41 (47.7)	20 (41.7)	21 (55.3)	χ^2^ = 1.5, *p* = 0.21
	Man	45 (52.3)	28 (58.3)	17 (44.7)	
Current age*		55.2 (15.1)	60.0 (10.0)	49.1 (18.1)	t = –3.5, *p* = 0.001
Years of education*		11.4 (4.2)	10.8 (4.2)	12.1 (4.1)	t = 1.4, *p* = 0.15
Current occupation*§*					
	Unemployed	25 (29.1)	18 (37.5)	7 (18.4)	χ^2^ = 3.9, *p* = 0.14
	Employed	37 (43.0)	19 (39.6)	18 (47.4)	
	Activity without remuneration	24 (27.9)	11 (22.9)	13 (34.2)	
Clinical variables					
Require help for daily activities*§*					
	No	52 (60.5)	38 (79.2)	14 (36.8)	χ^2^ = 15.8, *p * < 0.001
	Yes	34 (39.5)	10 (20.8)	24 (63.2)	
Hours dedicated to patient’s care*		9.2 (6.9)	–	9.2 (6.9)	–
MDS-UPDRS III*		28.3 (10.8)	28.3 (10.8)	–	–
PDQ-39*					
	Mobility	25.0 (19.2)	25.0 (19.2)	–	–
	Emotional well-being	19.6 (20.2)	19.6 (20.2)	–	–
	Stigma	15.1 (18.3)	15.1 (18.3)	–	–
	Cognition	20.9 (17.9)	20.9 (17.9)	–	–
	Social support	23.6 (26.6)	23.6 (26.6)	–	–
	Communication	21.3 (20.5)	21.3 (20.5)	–	–
	Bodily discomfort	28.3 (22.3)	28.3 (22.3)	–	–
	Summary Index	23.1 (13.5)	23.1 (13.5)	–	–
Zarit Burden Interview*		21.6 (17.1)	–	21.6 (17.1)	–

* Data presented in Means (SD); *§* n, sample size (number of observations); %, percentage; χ^2^, chi-square statistic; *p*, *p*-value (probability); t, Student’s *t* statistic; MDS-UPDRS III, Movement Disorder Society–Unified Parkinson’s Disease Rating Scale; Part III (Motor Examination); PDQ-39, Parkinson’s Disease Questionnaire (39-item).

### Psychiatric Symptoms, Cognition, and Internalized Stigma

No significant differences were found between PPD and PCG in the current severity of depressive and cognitive symptoms. Anxiety levels differed between groups (*p* = 0.008): PPD more frequently reported mild to moderate anxiety (mild: n = 21, 43.8%; moderate: n = 8, 16.7%), whereas PCG showed a higher proportion of severe anxiety. A higher proportion of PPD also exhibited mild cognitive impairment (n = 25, 52.1%), though this did not reach statistical significance. When comparing internalized stigma, there were no differences between the groups on the three scale dimensions or the total score (see Table 2).

**Table 2.** **Psychiatric symptoms and internalized stigma between patients with Parkinson’s disease and primary caregivers**.

		Total	Patients	Primary caregivers	Statistics
		n = 86	n = 48	n = 38	
Psychiatric symptoms					
PHQ-9 rating score*		3.1 (3.4)	3.3 (2.7)	2.8 (4.3)	t = –0.5, *p* = 0.56
Depression levels*§*					
	No	70 (81.4)	39 (81.3)	31 (81.6)	χ^2^ = 4.2, *p* = 0.23
	Mild	9 (10.5)	7 (14.6)	2 (5.3)	
	Moderate	6 (7.0)	2 (4.2)	4 (10.5)	
	Severe	1 (1.2)	-	1 (2.6)	
GAD rating score*		4.2 (4.3)	4.6 (3.7)	3.7 (5.0)	t = –0.9, *p* = 0.35
Anxiety levels*§*					
	No	41 (47.7)	16 (33.3)	25 (65.8)	χ^2^ = 11.7, *p* = 0.008
	Mild	27 (31.4)	21 (43.8)	6 (15.8)	
	Moderate	11 (12.8)	8 (16.7)	3 (7.9)	
	Severe	7 (8.1)	3 (6.3)	4 (10.5)	
MoCA (score)*		25.2 (3.1)	25.1 (3.2)	25.9 (3.1)	t = 0.3, *p* = 0.72
Mild cognitive impairment*§*					
	No	49 (57.0)	23 (47.9)	26 (68.4)	χ^2^ = 3.6, *p* = 0.056
	Yes	37 (43.0)	25 (52.1)	12 (31.6)	
King Internalized Stigma Scale					
Discrimination*		11.1 (7.7)	11.3 (8.2)	10.8 (7.0)	t = –0.3, *p* = 0.75
Disclosure*		10.5 (6.2)	10.6 (6.2)	10.3 (6.3)	t = –0.1, *p* = 0.85
Positive aspects*		5.1 (3.3)	5.5 (3.6)	4.6 (3.0)	t = –1.2, *p* = 0.22
Total score*		26.7 (13.7)	27.5 (14.2)	25.8 (13.2)	t = –0.5, *p* = 0.57

* Data presented in Means (SD); *§* n, sample size (number of observations); %, percentage; χ^2^, chi-square statistic; *p*, *p*-value (probability); t, Student’s *t* statistic; MoCA, Montreal Cognitive Assessment.

### Regression Analysis

For the linear regression model of PPD, the VIF values of the variables included in the model ranged from 1.20 to 2.43, indicating no multicollinearity. The backward conditional method indicated that variables associated with lower QoL were the severity of depressive, anxious, and PD symptoms. This means that a one-point increase in depression or anxiety severity was associated with a significant deterioration in patients’ QoL. In the same model, greater motor symptom severity, as measured by the MDS-UPDRS III, was also significantly associated with poorer QoL; although the magnitude of this effect was smaller (β = 0.39), it remained clinically meaningful.

For the PCG, VIF values ranged from 1.19 to 5.15 (no multicollinearity), and higher levels of depressive symptoms and greater perceived discrimination were significantly associated with increased caregiver burden (R^2^ = 0.53). Depressive symptoms showed a strong association with caregiver burden (β = 2.34, 95% CI 1.23–3.44, *p* = 0.001), while perceived discrimination was also significantly associated with greater burden (β = 0.63, 95% CI 0.12–1.39, *p* = 0.04). The remaining characteristics of both models are presented in Table 3.

**Table 3.** **Variables associated with quality of life in patients with Parkinson’s disease and burden in patients’ primary caregivers**.

	β coefficient	S.E.	95% C.I. β	*p*
Patients with Parkinson’ Disease				
Quality of life (R^2^ = 0.47)				
Depressive symptoms	1.50	0.63	0.23–2.77	0.02
Anxious symptoms	1.30	0.46	0.37–2.22	0.007
Motor symptoms (MDS-UPDRS III)	0.39	0.14	0.15–0.67	0.007
Primary caregivers				
Caregivers burden (R^2^ = 0.53)				
Depressive symptoms	2.34	0.53	1.23–3.44	0.001
Discrimination (internalized stigma scale)	0.63	0.36	0.12–1.39	0.04

β, beta (regression coefficient); SE, standard error (of β); 95% C.I. β, 95% confidence interval (for β); *p*, *p*-value; R^2^, coefficient of determination (variance explained by the model); MDS-UPDRS III, Movement Disorder Society–Unified Parkinson’s Disease Rating Scale, Part III (Motor Examination).

## Discussion

The present study aimed to determine the association between clinical characteristics (e.g., cognition, depression, and anxiety severity), sociodemographic factors, and stigma with QoL in PPD and the burden on PCG in the Mexican population. Unlike studies that focus on PPD or PCG alone, we assessed both members of the patient–caregiver dyad in the same clinical setting. This approach allowed us to examine how psychiatric symptoms and internalized stigma relate to patient QoL and caregiver burden within the same context.

In our study, PD affects more men than women, as recognized in the literature [43]. It is noteworthy to highlight that 34.2% of PCG were engaged in unpaid activities, including responsibilities related to primary caregiving.

Given that PD typically has a late onset, it is common for PPD to have comorbidities, with hypertension being prominent in our sample, which may complicate the care and management of PPD. The mean disease duration was 7.3 years, consistent with an early-to-mid stage cohort, which is also reflected in the MDS-UPDRS III scores [36, 38]. In addition, higher scores on the PHQ-9 and GAD-7 scales were associated with lower QoL in PPD. Depression and anxiety are known to be the most common psychiatric comorbidities in PPD, with 20% to 50% experiencing them at some stage of the disease, and they may even be present in the prodromal stage [44]. Identifying these symptoms can be challenging, as they overlap with motor and cognitive symptoms, potentially delaying pharmacological treatment, further compromising QoL in PPD, and increasing the burden on PCG [45].

Cognitive impairment is not typically expected in caregivers. However, just under a third of caregivers in our sample screened below the MoCA cutoff, which may reflect stress-related vulnerability rather than a primary neurodegenerative process; this should be examined in future studies [46].

As the disease progresses, PPD become less independent and require assistance from another person with ADLs, in particular a female caregiver, which is consistent with previous literature about the role of women in assistance activities [47, 48]. Role changes are common, as regardless of the familial relationship with the patient, it transforms into a caregiving role. While the mean score on the ZBI for PCG indicated a ‘low average’ burden, the wide range of scores (0–60) suggests considerable variability in individual experiences of burden. This finding, coupled with the identification of perceived discrimination and depression symptoms as significant factors associated with increased PCG burden, underscores that even if the average burden is not severe, specific factors can substantially impact a subset of PCG [48]. This reinforces the need for targeted interventions that identify and support those at higher risk. In addition, PCG may experience increased stress and burden as the disease progresses, highlighting the importance of providing healthcare team support based on the evolving needs during patient care. Addressing the needs of PCG can improve the care provided to PPD and benefit their QoL [49]. The discrepancy between PCG and PPD perceptions of care needs is striking. PCG who report providing extended daily care may be attending to PPD with more advanced disease stages. Alternatively, PPD may not realize that their care is burdensome to PCG, as has been reported in other studies [50, 51].

Depressive and anxiety symptoms in PPD have a significant effect on their QoL. These symptoms, along with the clinical burden of the disease, contribute to a lower perception of well-being and functionality across multiple domains, as assessed by the PDQ-39. These symptoms can also complicate treatment adherence and therapeutic response, exacerbating the negative impact on QoL. Beyond the comparative analyses between groups, it is clinically salient to highlight the high prevalence of anxiety symptoms within our cohort, affecting approximately two-thirds of PPD (66.8% with mild to severe anxiety) and over one-third of PCG (34.2% with mild to severe anxiety). Similarly, depressive symptoms were present in a notable proportion of both groups (18.8% of PPD and 18.4% of PCG exhibiting mild to severe symptoms) and were directly associated with a diminished QoL in PPD and caregivers’ burden. These data underscore the critical need for routine mental health screening and proactive management in both PPD and PCG, irrespective of whether group differences reach statistical significance on all measures.

Evaluating the causes and factors associated with the presence of depression in both PPD and PCG is highly important, as depression is a multifactorial condition influenced by clinical, psychological, and social variables that directly affect QoL, treatment adherence, and caregiving burden. Although differences in anxiety severity were observed, examining the underlying factors contributing to these differences was beyond the scope and objectives of the present study. Addressing these factors in future research will be vital for implementing targeted treatment strategies to improve the mental health of both groups.

Additionally, while no significant differences were found in the mean internalized stigma scores between PPD and PCG, our regression analysis revealed that greater perceived discrimination (a subdomain of internalized stigma) was significantly associated with increased PCG burden. This highlights that even if average stigma levels do not differ between groups, specific facets of internalized stigma can still act as factors associated with adverse outcomes within a group [52, 53], such as PCG burden.

Maintaining proper symptom control in PD, including rehabilitation to improve speech and gait, is critical to preserving independence in ADLs for as long as possible. This also helps PCG cope with the burden of caring for someone with a neurodegenerative disease. Ongoing assessment of depressive and anxiety symptoms is also important in clinical practice, as effective management of these symptoms contributes to improved QoL for both PPD and their PCG. For health services to address the needs arising from a disease, they must understand the complex biopsychosocial framework of PD, based on comprehensive knowledge of the biological, psychological, and social aspects of the disease. Globally, and particularly in Mexico, there is a need for more knowledge and research on internalized stigma in neurodegenerative diseases, as it can negatively impact QoL perception and how PPD and their PCG face the disease, as well as their decision-making. This is an important aspect to consider in public health policies. A study reported that early age of onset of a disease and the presence of depression are major factors influencing high levels of internalized stigma [20]. Therefore, internalized stigma affects how a patient perceives their QoL.

A notable strength of this study is the substantial explanatory power of our regression models, which accounted for more than 40% of the variance in both patient QoL and PCG burden. This indicates that the identified psychosocial and clinical factors (depressive symptoms, PD severity, perceived discrimination) are highly relevant and significant contributors to the overall well-being of these populations. This finding further emphasizes the importance of integrating these variables into comprehensive clinical assessments and the development of targeted interventions.

This study has several limitations. The modest sample size (48 PPD and 38 PCG) reduces statistical power, limits within-group analyses, and may constrain generalizability to the wider Mexican PD population. Group sizes differed because some patients attended visits without a caregiver; accordingly, regression models were conducted separately for patients and caregivers. As participants did not represent the full range of PD stages, findings may not extend to very early or advanced disease. Detailed medication data (dose, duration, and specific regimens) were not collected, and treatment-related factors may have influenced some associations. Socioeconomic status (SES) was inconsistently recorded for PPD and was unavailable for PCG, so it was not included in Table 1 or in inferential analyses. Finally, several outcomes relied on self-reported instruments, which may introduce reporting bias and could partly explain discrepancies between patient and caregiver reports of assistance with activities of daily living. Larger, longitudinal studies including a broader range of disease stages and more complete contextual data are needed to clarify the direction and stability of these associations.

Future research should aim to recruit a more diverse sample that spans the full spectrum of disease stages to provide a comprehensive understanding of how these factors influence QoL and PCG burden throughout the entire course of PD. It is also essential to consider the impact of cultural context on how diseases such as Parkinson’s disease is perceived, interpreted, and managed, as cultural beliefs and social norms shape attitudes toward symptoms, disability, and caregiving. Accordingly, our findings, which may be considered preliminary, are likely influenced by this cultural specificity and should be interpreted within the sociocultural context in which the study was conducted.

Although this study identifies factors significantly associated with QoL and PCG burden, it is crucial to acknowledge the cross-sectional nature of its design. This limits our ability to establish causal relationships, meaning we cannot determine if these identified factors directly cause the observed outcomes or if the relationships are bidirectional. Future longitudinal studies with a significantly larger sample are essential to elucidate the temporality and causality of these associations, further enriching the international discourse on PD management and developing tailored interventions within the unique cultural contexts of the Mexican population.

## Conclusions

This study characterizes the dynamics and challenges faced by PPD and their PCG in Mexico. Among PPD, poorer QoL was independently associated with depressive symptoms, anxiety symptoms, and motor severity (MDS-UPDRS III). Among PCG, a greater burden was independently associated with depressive symptoms and perceived discrimination (internalized stigma subdomain). Although PCG were younger than PPD, age was not an independent predictor and did not alter these associations in multivariable models. These findings support dyad-focused interventions that combine caregiver psychoeducation with targeted mental health care to address internalized stigma and psychiatric symptoms, thereby improving QoL and reducing PCG burden.

## Availability of Data and Materials

The datasets and materials used and/or analyzed during the current study are available from the corresponding author upon reasonable request.

## References

[1] Florijn BW, Kloppenborg R, Kaptein AA, Bloem BR (2023). Narrative medicine pinpoints loss of autonomy and stigma in Parkinson’s disease. *NPJ Parkinson’s Disease*.

[2] Luo Y, Qiao L, Li M, Wen X, Zhang W, Li X (2025). Global, regional, national epidemiology and trends of Parkinson’s disease from 1990 to 2021: findings from the Global Burden of Disease Study 2021. *Frontiers in Aging Neuroscience*.

[3] Riccò M, Vezzosi L, Balzarini F, Gualerzi G, Ranzieri S, Signorelli C (2020). Prevalence of Parkinson Disease in Italy: a systematic review and meta-analysis. *Acta Bio-Medica: Atenei Parmensis*.

[4] Institute for Health Metrics and Evaluation (2025). GBD Results Tool. Institute for Health Metrics and Evaluation. https://vizhub.healthdata.org/gbd-results/.

[5] Pereira GM, Teixeira-Dos-Santos D, Soares NM, Marconi GA, Friedrich DC, Saffie Awad P (2024). A systematic review and meta-analysis of the prevalence of Parkinson’s disease in lower to upper-middle-income countries. *NPJ Parkinson’s Disease*.

[6] Lazaro-Figueroa A, Reyes-Perez P, Morelos-Figaredo E, Guerra-Galicia CM, Estrada-Bellmann I, Salinas-Barboza K (2023). Clinical and genetic insights of Parkinson’s Disease in a Mexican cohort: highlighting Latino’s diversity. *medRxiv*.

[7] Sveinbjornsdottir S (2016). The clinical symptoms of Parkinson’s disease. *Journal of Neurochemistry*.

[8] Weintraub D, Mamikonyan E (2019). The Neuropsychiatry of Parkinson Disease: A Perfect Storm. *The American Journal of Geriatric Psychiatry*.

[9] Cavanagh JF, Ryman S, Richardson SP (2022). Cognitive control in Parkinson’s disease. *Progress in Brain Research*.

[10] Ortiz G, Gonzalez-Usigli H, Pacheco-Moisés F, Velázquez-Brizuela I, Sánchez-López A, González E (2020). Gender Differences in Patients with Depression and Anxiety with Parkinson’s Disease. *Archivos de Neurociencias*.

[11] Islam SS, Neargarder S, Kinger SB, Fox-Fuller JT, Salazar RD, Cronin-Golomb A (2022). Perceived stigma and quality of life in Parkinson’s disease with additional health conditions. *General Psychiatry*.

[12] Chiong-Rivero H, Ryan GW, Flippen C, Bordelon Y, Szumski NR, Zesiewicz TA (2011). Patients’ and caregivers’ experiences of the impact of Parkinson’s disease on health status. *Patient Related Outcome Measures*.

[13] Balash Y, Korczyn AD, Knaani J, Migirov AA, Gurevich T (2017). Quality-of-life perception by Parkinson’s disease patients and caregivers. *Acta Neurologica Scandinavica*.

[14] Vásquez L, Tamariz-Rodriguez A, Gutiérrez Pérez JR, Marín G, Toledo R, Carrillo P (2019). Parkinson’s Disease Beyond Motor Symptoms. *eNeurobiología*.

[15] Rajiah K, Maharajan MK, Yeen SJ, Lew S (2017). Quality of Life and Caregivers’ Burden of Parkinson’s Disease. *Neuroepidemiology*.

[16] Mula M, Sander JW (2016). Psychosocial aspects of epilepsy: a wider approach. *BJPsych Open*.

[17] Fung KMT, Tsang HWH, Chan F (2010). Self-stigma, stages of change and psychosocial treatment adherence among Chinese people with schizophrenia: a path analysis. *Social Psychiatry and Psychiatric Epidemiology*.

[18] Mehanna R (2020). “I am not depressed. I just don’t like myself”: stigma and (lack of) depression in a subset of patients with Parkinson’s disease. *Revista Brasileira De Psiquiatria*.

[19] Ma HI, Saint-Hilaire M, Thomas CA, Tickle-Degnen L (2016). Stigma as a key determinant of health-related quality of life in Parkinson’s disease. *Quality of Life Research*.

[20] Masoudi R, Khayeri F, Rabiei L, Zarea K (2017). A study of stigma among Iranian family caregivers of patients with multiple sclerosis: A descriptive explorative qualitative study. *Applied Nursing Research*.

[21] Chou PC, Lee Y, Chang YY, Hung CF, Chen YF, Lin TK (2024). The Interrelationship of Benefit Finding, Demoralization, and Stigma among Patients with Parkinson’s Disease and Their Caregivers. *Healthcare*.

[22] Brydges CR (2019). Effect Size Guidelines, Sample Size Calculations, and Statistical Power in Gerontology. *Innovation in Aging*.

[23] Postuma RB, Berg D, Stern M, Poewe W, Olanow CW, Oertel W (2015). MDS clinical diagnostic criteria for Parkinson’s disease. *Movement Disorders*.

[24] Postuma RB, Poewe W, Litvan I, Lewis S, Lang AE, Halliday G (2018). Validation of the MDS clinical diagnostic criteria for Parkinson’s disease. *Movement Disorders*.

[25] Peto V, Jenkinson C, Fitzpatrick R, Greenhall R (1995). The development and validation of a short measure of functioning and well being for individuals with Parkinson’s disease. *Quality of Life Research*.

[26] Martínez-Martín P, Frades Payo B (1998). Quality of life in Parkinson’s disease: validation study of the PDQ-39 Spanish version. The Grupo Centro for Study of Movement Disorders. *Journal of Neurology*.

[27] Julayanont P, Nasreddine Z, Larner AJ (2017). Montreal Cognitive Assessment (MoCA): Concept and Clinical Review. *Cognitive Screening Instruments*.

[28] Aguilar-Navarro SG, Mimenza-Alvarado AJ, Palacios-García AA, Samudio-Cruz A, Gutiérrez-Gutiérrez LA, Ávila-Funes JA (2018). Validity and Reliability of the Spanish Version of the Montreal Cognitive Assessment (MoCA) for the Detection of Cognitive Impairment in Mexico. *Revista Colombiana De Psiquiatria*.

[29] Spitzer RL, Kroenke K, Williams JB (1999). Validation and utility of a self-report version of PRIME-MD: the PHQ primary care study. Primary Care Evaluation of Mental Disorders. Patient Health Questionnaire. *JAMA*.

[30] Spitzer RL, Kroenke K, Williams JBW, Löwe B (2006). A brief measure for assessing generalized anxiety disorder: the GAD-7. *Archives of Internal Medicine*.

[31] Familiar I, Ortiz-Panozo E, Hall B, Vieitez I, Romieu I, Lopez-Ridaura R (2015). Factor structure of the Spanish version of the Patient Health Questionnaire-9 in Mexican women. *International Journal of Methods in Psychiatric Research*.

[32] Arrieta J, Aguerrebere M, Raviola G, Flores H, Elliott P, Espinosa A (2017). Validity and Utility of the Patient Health Questionnaire (PHQ)-2 and PHQ-9 for Screening and Diagnosis of Depression in Rural Chiapas, Mexico: A Cross-Sectional Study. *Journal of Clinical Psychology*.

[33] García-Campayo J, Zamorano E, Ruiz MA, Pardo A, Pérez-Páramo M, López-Gómez V (2010). Cultural adaptation into Spanish of the generalized anxiety disorder-7 (GAD-7) scale as a screening tool. *Health and Quality of Life Outcomes*.

[34] King M, Dinos S, Shaw J, Watson R, Stevens S, Passetti F (2007). The Stigma Scale: development of a standardised measure of the stigma of mental illness. *The British Journal of Psychiatry*.

[35] Flores Reynoso S, Medina Dávalos R, Robles García R (2011). Spanish Translation Study and Psychometric Evaluation of a Scale to Measure Internalized Stigma in Patients with Severe Mental Disorders. *Salud Mental*.

[36] Goetz CG, Tilley BC, Shaftman SR, Stebbins GT, Fahn S, Martinez-Martin P (2008). Movement Disorder Society-sponsored revision of the Unified Parkinson’s Disease Rating Scale (MDS-UPDRS): scale presentation and clinimetric testing results. *Movement Disorders*.

[37] Martínez-Martín P, Rodríguez-Blázquez C, Mario Alvarez, Arakaki T, Arillo VC, Chaná P (2015). Parkinson’s disease severity levels and MDS-Unified Parkinson’s Disease Rating Scale. *Parkinsonism & Related Disorders*.

[38] Holden SK, Finseth T, Sillau SH, Berman BD (2018). Progression of MDS-UPDRS Scores Over Five Years in De Novo Parkinson Disease from the Parkinson’s Progression Markers Initiative Cohort. *Movement Disorders Clinical Practice*.

[39] Zarit SH, Reever KE, Bach-Peterson J (1980). Relatives of the impaired elderly: correlates of feelings of burden. *The Gerontologist*.

[40] Galindo-Vazquez O, Benjet C, Cruz-Nieto MH, Rojas-Castillo E, Riveros-Rosas A, Meneses-Garcia A (2015). Psychometric properties of the Zarit Burden Interview in Mexican caregivers of cancer patients. *Psycho-Oncology*.

[41] Alshammari SA, Alzahrani AA, Alabduljabbar KA, Aldaghri AA, Alhusainy YA, Khan MA (2017). The burden perceived by informal caregivers of the elderly in Saudi Arabia. *Journal of Family & Community Medicine*.

[42] de Bie RMA, Katzenschlager R, Swinnen BEKS, Peball M, Lim SY, Mestre TA (2025). Update on Treatments for Parkinson’s Disease Motor Fluctuations - An International Parkinson and Movement Disorder Society Evidence-Based Medicine Review. *Movement Disorders*.

[43] Cerri S, Mus L, Blandini F (2019). Parkinson’s Disease in Women and Men: What’s the Difference?. *Journal of Parkinson’s Disease*.

[44] Heim B, Djamshidian A (2025). Neuropsychiatric disorders in Parkinson’s disease. *Therapeutic Advances in Neurological Disorders*.

[45] Weintraub D, Aarsland D, Chaudhuri KR, Dobkin RD, Leentjens AF, Rodriguez-Violante M (2022). The neuropsychiatry of Parkinson’s disease: advances and challenges. *The Lancet. Neurology*.

[46] Vitaliano PP, Murphy M, Young HM, Echeverria D, Borson S (2011). Does caring for a spouse with dementia promote cognitive decline? A hypothesis and proposed mechanisms. *Journal of the American Geriatrics Society*.

[47] Martinez-Martin P, Arroyo S, Rojo-Abuin JM, Rodriguez-Blazquez C, Frades B, de Pedro Cuesta J (2008). Burden, perceived health status, and mood among caregivers of Parkinson’s disease patients. *Movement Disorders*.

[48] Kumar H, Ansari S, Kumar V, Barry HD, Tahir A (2019). Severity of Caregiver Stress in Relation to Severity of Disease in Persons with Parkinson’s. *Cureus*.

[49] Martinez-Martin P, Skorvanek M, Henriksen T, Lindvall S, Domingos J, Alobaidi A (2023). Impact of advanced Parkinson’s disease on caregivers: an international real-world study. *Journal of Neurology*.

[50] Perepezko K, Hinkle JT, Forbes EJ, Pontone GM, Mills KA, Gallo JJ (2023). The impact of caregiving on quality of life in Parkinson’s disease: A systematic review. *International Journal of Geriatric Psychiatry*.

[51] May CR, Gravenhorst KC, Hillis A, Arber M, Chew-Graham CA, Gallacher KI (2025). How lived experiences of illness trajectories, burdens of treatment, and social inequalities shape service user and caregiver participation in health and social care: a theory-informed qualitative evidence synthesis. *Health and Social Care Delivery Research*.

[52] Maffoni M, Giardini A, Pierobon A, Ferrazzoli D, Frazzitta G (2017). Stigma Experienced by Parkinson’s Disease Patients: A Descriptive Review of Qualitative Studies. *Parkinson’s Disease*.

[53] Karacan AV, Kibrit SN, Yekedüz MK, Doğulu N, Kayis G, Unutmaz EY (2023). Cross-Cultural Differences in Stigma Associated with Parkinson’s Disease: A Systematic Review. *Journal of Parkinson’s Disease*.

